# The well-worn path from armed conflict to measles resurgence

**DOI:** 10.1371/journal.pmed.1005156

**Published:** 2026-06-26

**Authors:** José E. Hagan

**Affiliations:** US Centers for Disease Control and Prevention, Global Health Center, Global Immunization Division, Atlanta, Georgia, United States of America

## Abstract

In this Perspective, José Hagan discusses how and why weakened immunization, surveillance, and socioeconomic resilience due to armed conflict increases measles risk during and after war, and outlines why protecting routine vaccination in crises should be a core emergency response.

## Teaser

Armed conflict weakens immunization, surveillance, and socioeconomic resilience, increasing measles risk during and after war. Protecting routine vaccination in crises is a core emergency response.

Among the many sequelae of war is a legacy of damaged health systems and the resurgence of outbreak-prone diseases normally held at bay by functioning national vaccination programs. Today, 20% of the global birth cohort lives in countries affected by conflict and instability [1]; yet, these countries account for more than half of the world’s 14.5 million “zero-dose” children [2]. These are infants and young children who did not receive a single dose of the three-dose diphtheria–tetanus–pertussis (DTP) vaccine series, a marker for the most basic failure of access to immunization services.

As the most infectious and outbreak-prone disease, a single measles case can generate up to 18 additional infections, with infectious virus particles remaining airborne and on surfaces for up to two hours [3,4]. To maintain herd immunity and prevent widespread measles transmission, immunization programs need to maintain at least 95% vaccination coverage. Measles virus transmission therefore provides a sensitive stress test of health system functioning, with outbreaks easily exposing even brief or localized interruptions in routine immunization, surveillance, and access to services [5,6]. Measles epidemics have been a major cause of mortality in conflict-affected populations, with historical examples showing measles accounted for 53% and 42% of deaths in conflict refugees in eastern Sudan and Somalia in 1985, respectively [7]. Further, malnutrition, a risk in conflict areas, sharply increases measles mortality; malnutrition contributes to death in 45% of fatal measles cases, and case fatality can reach as high as 33% in complex emergency settings [7].

Conflict and insecurity can directly disrupt routine immunization through facility destruction, supply chain breakdowns, and loss of health workers; and degradation of surveillance and laboratory functions delays detection and response, increasing the probability that small outbreaks become large. These dynamics have been repeatedly documented across conflict settings; a recent systematic review found most empirical studies reported declines in vaccination coverage during armed conflict [8]. In a recent *PLOS Medicine* study [9], Tozan and Headley analyzed longitudinal data from 193 countries during 2000–2023 using fixed-effects panel regression structural equation modeling (SEM) to explore the direct relationship between war (measured as battle-related deaths) and measles cases, and test an exploratory pathway linking armed conflict, displacement, socioeconomic development, and measles burden.

The SEM analysis used in this study suggests an indirect association between conflict and measles burden through weakened socioeconomic and health system resilience. In the authors’ framework, armed conflict erodes the socioeconomic structures that support public health (measured as a composite of GDP per capita, life expectancy, and education), which in turn is associated with higher measles burden. The model results also highlight conflict-related mass population displacement in this indirect pathway, which places further strain on and further degrades these same socioeconomic structures, driving broader conditions that support measles spread. We know that displacement itself often leads to overcrowding and population mixing in shelters or camps, which increases infectious disease transmission opportunities, especially measles [7,10].

In the short term, conflict can rapidly reduce coverage among infants and young children through interrupting service access and delivery, while displacement compresses an increasingly measles-susceptible population into high-contact settings. The authors’ analysis includes a time component, estimating a long tail of delayed association between conflict and measles burden, that peaks around 3 years later. Persistent insecurity, disrupted schooling, reduced household income, and weakened governance can further entrench immunity gaps, creating a rapidly growing pool of unvaccinated children who are susceptible to measles, leading to sometimes explosive outbreaks months or years later [8,11].

The authors interpret their findings with appropriate caution. Conflict causes an external shock to health systems, but at the same time, as the authors acknowledge, conflict is often not an external force but emerges locally from the same conditions that contribute to the risk of outbreaks: local institutional fragility, poverty, political instability, and exclusion of marginalized populations. Crises such as large-scale outbreaks can themselves exacerbate socioeconomic instability and heighten social tensions between groups, potentially reinforcing the dynamics that lead to internal conflict. Although the authors reported that models testing reverse pathways from measles incidence to conflict and displacement performed poorly, the findings still sit within a complex and reciprocal relationship between conflict, displacement, economic development, and public health, rather than a single causal pathway from conflict to health outcomes.

There are central analytic challenges in this field of research in that the same structural conditions that shape conflict may also shape health system resilience, surveillance performance, displacement, and outbreak risk, and in the inherent uncertainties of the available data. The authors’ analysis uses national-level data, which risks dissolving localized conflicts into the national aggregate; and data drawn from surveillance systems interrupted or weakened by war will unsurprisingly undercount measles cases in the most affected geographies. These limitations mean that the authors’ estimates of association between war and measles cases are likely conservative, and underscore a broader methodological challenge in conflict and health research. The populations suffering the greatest violence may also be the most epidemiologically invisible.

Measles has been described as a ‘tracer’ of immunization system strengths and weaknesses, where measles cases signal the failure of the health system to reach specific populations [5]. Measles appears early because it is so transmissible and its prevention is so dependent on uninterrupted immunization. Following war, measles outbreaks may be a sentinel event for a broader infectious disease aftershock. Conflict-related disruption has also been linked to infectious disease outbreaks beyond measles [12]. Crowding, malnutrition, interrupted care, displacement, weakened governance, and surveillance failure are not only potential drivers of measles, but are potential pathways from conflict to contagion more broadly.

This study supports and reinforces global guidance: In conflict and emergency settings, protecting, sustaining and adapting the basic architecture of routine vaccination and disease surveillance is itself an emergency response [13]. This involves deliberate and tailored inclusion of displaced populations in routine immunization and surveillance from the outset. These basic functions cannot be relegated to a secondary service to be rebuilt during future reconstruction after addressing more visibly urgent competing priorities of trauma care, mental health, emergency nutrition, warmth, and shelter. The toll of war is not only measured in violent deaths but also in the preventable illnesses and deaths that follow. Reducing war’s impact on prolonged human suffering through infectious diseases necessitates a commitment to preserve and rebuild immunization at speed and scale, and to protect the economic and health systems capacities that make high population immunity durable.

## Abbreviations

DTP: diphtheria–tetanus–pertussis
SEM: structural equation modeling
